# Spatial heterogeneity of soil nutrients in Yili River Valley

**DOI:** 10.7717/peerj.13311

**Published:** 2022-05-03

**Authors:** Guojun Sun, Haijun Liu, Dong Cui, Chunmei Chai

**Affiliations:** 1 School of Environmental Law, Gansu University of Political Science and Law, Lanzhou, Gansu, China; 2 College of Biological and Geographical Sciences, Yili Normal University, Yining, Xinjiang, China; 3 The College of Life and Geographic Sciences, Kashgar University, Kashgar, Xinjiang, Kashgar, China

**Keywords:** Soil nutrient, Spatial variability, Geostatistics, Yili River Valley

## Abstract

Soil nutrients are a vital reference index of soil fertility and are essential in studying spatial variability for the development of land resources. The traditional statistical methods including correlation analysis and geostatistical analysis, were used to explore the spatial variability of nutrients and its influencing factors in the Yili River Valley. The results showed that soil total potassium (STK) had a weak variation, soil organic carbon (SOC), soil total nitrogen (STN) and soil total phosphorus (STP) showed a moderate degree of variation. Correlation analysis showed that SOC had a significant correlation with STN, STP, STK, silt, soil water content (SWC), Cos a and altitude (*p* < 0.01). In contrast, negative correlations were found between the SOC and sand, soil bulk density (SBD) and pH (*p* < 0.01), the same as STN. STP had a significant correlation with STK, silt (*p* < 0.01) and Cos a (*p* < 0.05), whereas negative correlations were found between the STP, sand and SBD (*p* < 0.01). STK had a significant correlation with silt, whereas negative correlations were found between the STK, sand and SBD. Ordinary Kriging interpolation showed that the distribution of SOC and STN had a high value in the northeast, northwest and southeast, and a low value in the central and southwest. STP was high in the northwest and southeast and low in the northeast and southwest. STK was high in the northwest and northeast and low in the central and southeast regions. This is helpful for the rational exploitation of land resources in ecological economy development in the Yili River Basin.

## Introduction

Soil plays an essential role in the biogeochemical cycle, and it is an important for the source and depositions of soil nutrients (Zhao et al., 2009). Soil is naturally complex and affected by both natural and human factors. There is also strong spatial heterogeneity in soil (Zhao et al., 2011a) meaning that within a certain landscape, at the same time and in different locations, there are obvious differences and diversities in soil properties (Wang et al., 2014). Soil fertility is one of the most important ecological functions of the soil (Cui, Gao & Lü, 2010). Soil nutrients are an important factor in measuring the level of soil fertility, and are also the most important factor in vegetation growth and reproduction (Yang et al., 2013; Jing et al., 2014). Studying spatial variability and exploring spatial evolution law is important as these have theoretical and practical significance in the rational development and utilization of land resources (Zhao et al., 2007).

Since the 1970s, the variation of soil nutrients has been an important topic in soil science research (Jing et al., 2014; Zhang et al., 2014). Soil heterogeneity promoted plant species coexistence and plant species diversity. Wei (2019) showed that high nutrient soil patches supported higher species richness and community biomass. Heterogeneity in soil nutrient supply reduced plant species diversity (Gazol et al., 2013). Statistical methods were used to study soil nutrient changes that have become more popular. Traditional studies on nutrient variability used qualitative research methods and statistics (Fu et al., 1999), but it was difficult to reflect the spatial changes of soil nutrients and explain spatial heterogeneity. The advantages of geostatistical methods have made up for the deficiencies of traditional statistical methods in the research of spatial difference, therefore the geostatistical methods have been widely used in the study of soil nutrient heterogeneity (Zhang et al., 2013a). The ordinary kriging interpolation method, an important spatial interpolation method in geo-statistics, was the main method used in this study of soil nutrient variability, which was able to predict the unknown sampling area by using the semi variance function and the optimal linear unbiased estimation (Zhang et al., 2010).

The Yili River Valley is the most potential agricultural reclamation area and the key control region of water erosion in the northwest of Xinjiang Uygur Autonomous Region (Sun et al., 2017). Studying the spatial variation of soil nutrients is important to ensure the success of the development and utilization of regional land resources. In the Yili River Valley, previous studies have shown that organic matter and total nitrogen included in the soil were positively correlated with slope, whereas total phosphorus was negatively correlated with the slope direction (Liu & Zhang, 2012), The soil organic carbon content showed basically a tendency to decrease as soil depth increases under various vegetation types except in the case of the intrazonal vegetation (Yang et al., 2010). At present, classical statistical methods have been used to study the variation characteristics of soil nutrients on a small scale (Liu, Shao & Wang, 2012; Li et al., 2010), but research on soil nutrient heterogeneity for large scale analysis is still rare. In this study, a traditional statistical method was used to analyze soil physical and chemical properties, classical geo-statistical methods and geo-statistical methods were used to study spatial variation characteristics, distribution and heterogeneity of soil nutrients in the area. The purpose of this study was to explore the formation mechanism and the reasons for change, thus providing a theoretical reference for the cultivated Yili Valley hillside, but also to provide theoretical support for the rational exploitation of land resources and the sustainable development of an ecological economy in the Yili River Basin.

## Materials and Methods

### Study area

**Figure 1:**
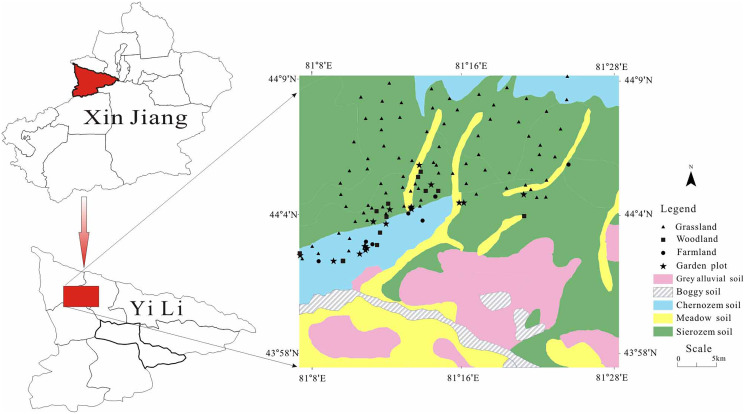
Map of the study area.

The Yili River Valley Basin is located on the western slope of the Tianshan Mountains southwest of the Zhungeer Basin, at latitude 42°14′–44°50′ and longitude 80°09′–84°56′. The Yili River Valley Basin is a rift basin separated by the south and north Tianshan mountains. These mountains surround the basin on the east, south, and north sides. From west to east, the elevation and rainfall gradually increase, the area has a typical temperate continental climate with annual average precipitation was 417.6 mm, an annual average temperature of 10.4 degrees Celsius, and 2,898.4 h of annual sunshine on average (Sun, Li & Yang, 2012). This area is about 5.53 × 10^4^ km^2^ in size; the distance from east to west is 360 km, and the length from south to north is 275 km (Yang et al., 2010). The sampling points were: 43°51′–44°9′N and 81°32′–81°28′E, in the north slope of Yining City (Fig. 1). The natural vegetation is temperate grassland vegetation and semi-desert grassland vegetation, and the soil type is mainly gray alluvial soil, boggy soil, chernozem soil, sierozem soil and meadow soil.

### Sample collection and analysis

We chose representative samples in the Yili River Valley in May 2015, associated with regional DEM, land use types, climate, soil and other natural conditions. The self-made sampler (10 cm diameter and 50 cm height) was used to collect topsoil samples (0–20 cm). Each sample point was positioned by GPS, and factors such as latitude, longitude and elevation were recorded. One hundred twenty-seven topsoil samples at depths of 0–20 cm were collected (Fig. 1) including cultivated land samples (nine), garden samples (18), woodland samples (12), and grassland samples (88). The organic carbon concentration of the soil (g•kg^−1^) was determined using the K_2_Cr_2_O_7_-external heating method; total nitrogen concentration (g•kg^−1^) was determined using the perchloric acid-sulphuric acid digestion method; total phosphorus in the soil was found using the acid-soluble and molybdenum antimony ratio colorimetric method; total potassium in the soil was determined using the acid digestion and flame photometric method; soil pH was determined using the potential method; SBD was determined using the ring shear testing method; water content was determined using oven drying method (Liu et al., 2016); and the Mastersizer 2000 particle size analyzer (Malvern Instruments, Malvern, England) was used to measure soil particle composition (Zhang et al., 2016).

## Methods

The SPSS19.0 software was used for the classical statistical analysis of the soil nutrient data, the ArcGIS10.0 software was used for the ordinary Kriging interpolation.

The semi variance function objectively depicts the degree of correlation between variables, if the distance between the two variables is close, the similarity is strong, and the semi variation function is small. If the distance between the two variables is far, the similarity is weak, and the semi-variation function is large (Zhao et al., 2007). The semivariance function can be calculated using the following formula:

$r(h) = \displaystyle{1 \over {2\left| {N(h)} \right|}}\sum\limits_{i = 1}^{N(h)} {\left[ {Z({{\rm x}_i}) - Z({x_i} + {\rm h})} \right]} \matrix{ 2 \cr {} \cr }$

where, *h* is the step size, *r*(*h*) is the semivariance function, *Z*(*x*) and *Z*(*x* + *h*) is the value of the regional variable at the point *x* and *x* + *h*, and *r*(*h*) is half of the variance of the difference between *Z*(*x*) and *Z*(*x* + *h*). In the semivariance function, when the sampling point distance is 0, the semi-variogram value should be 0. When the sampling point is very close but not quite zero, meaning the variogram is also not 0, a nugget (*C*_0_) is created. The semivariance function also increases when the distance of the sampling point increases. When the sampling point distance increases to a certain value, semi-variogram values flatten out to a constant (Sill). The difference between the base station value and the nugget value is called the partial base value (Cs). For the same region variable, the larger the value of the base station, the stronger the spatial correlation; when the regional variable is not the same, the value of the partial base station cannot reflect the spatial variability of the regional variables. The ratio of the partial base station and the base station value (Cs/Sill) can reveal the spatial correlation degree of the different regional variables: the greater the value, the stronger the spatial correlation. When the value is less than 25%, it has a weak spatial correlation; when the value is 25% to 75%, it has a moderate spatial correlation; and when the value is 75% to 100%, it has a strong spatial correlation (Zhao et al., 2007; Zhao et al., 2011a).

The Ordinary Kriging interpolation method is based on the theory of semi variance function and structural analysis. In a limited area, this method is one of the most effective methods for an unbiased estimate of the regional variables, which are spatially correlated (Zhao et al., 2011b). The equation for is the Ordinary Kriging interpolation method is as follows:

$Z({{\rm x}_0}) = \sum\limits_{i = 1}^n {{\lambda_i}} Z({x_{\rm i}})$

where *Z*(*x*_0_) is the value of the unknown sampling point, *Z*(*x*_i_) is the value of the known sampling point around the unknown sampling points, λ_i_ is the weight of the sampling point to the unknown sampling point, *n* is the number of known sample points.

## Results

### Classical statistical description of the physical and chemical properties of the soil

The coefficient of variation (CV) values were 68%, 57.76%, 12.62%, 28.99%, 13.51% and 49.74% for SOC, STN, STP, sand, silt and SWC, respectively, that ranged from 12.62% to 68.00%, indicating a moderate degree of variation. CV values were 9.17%, 8.82% and 1.89% for SBD, STK and pH, respectively, ranging from 1.89% to 9.17%, corresponding to a weak variability. SOC was between 2.04 g·kg^−1^ and 34.79 g·kg^−1^ with a mean value of 6.99 g·kg^−1^, which was lower than the Chinese national average value of 17.53 g·kg^−1^ (Li et al., 2010). STN values were between 0.24 g·kg^−1^ and 3.52 g·kg^−1^ with an average value of 0.79 g·kg^−1^, which was also lower than the Chinese national average value of 1.54 g·kg^−1^ (Li et al., 2010) (Table 1).

**Table 1:** Descriptive statistics for the topsoil physical and chemical properties.

Variable	Minimum	Maximum	Mean	SD	CV	DT
SOC	2.04	34.79	6.99	4.76	68	LND
STN	0.24	3.52	0.79	0.45	57.76	LND
STP	0.38	1.05	0.71	0.09	12.62	ND
STK	5.21	15.92	13.51	1.19	8.82	ND
Sand	14.06	69.07	32.39	9.39	28.99	ND
Silt	27.93	75.8	60.47	8.17	13.51	ND
SWC	0.2	12.44	4.99	2.48	49.74	LND
SBD	0.93	1.67	1.26	0.12	9.17	ND
pH	7.61	8.78	8.18	0.15	1.89	ND

**Note:**

SOC, Soil organic carbon (g·kg^−1^); STN, Soil total nitrogen (g·kg^−1^); STP, Soil total phosphorus (g·kg^−1^); STK, Soil total potassium (g·kg^−1^); SWC, Soil water content; SBD, Soil bulk density (g·cm^−3^); Sand (%); Silt (%); SD, Standard deviation; CV, Coefficient of variation (%); DT, Distribution type; LND, Log-normal distribution; ND, Normal distribution.

A non-parameter and single sample test of K-S showed that, SOC, SWC and STK values followed a normal distribution; STP, STK, sand, silt, SBD and pH values followed log-normal distribution (Table 1).

### Correlation analyses

The Pearson’s correlation coefficient was calculated to characterize the relationship between soil nutrients (SOC, STN, STP and STK) and selected properties (sand, silt, SWC, SBD and pH). SOC, STN, STP and STK had a significant correlation with silt content and a negative correlation with both sand and SBD. SWC was significantly positively correlated with SOC and STN, but had no relation to STP and STK. pH was significantly negatively correlated with SOC and STN, but had no relation to STP and STK (Table 2).

**Table 2:** The relationship between soil nutrients and selected properties.

	SOC	STN	STP	STK	Sand	Silt	SWC	SBD	pH
SOC	1								
STN	0.96**	1							
STP	0.33**	0.37**	1						
STK	0.24**	0.24**	0.37**	1					
Sand	−0.24**	−0.27**	−0.32**	−0.34**	1				
Silt	0.28**	0.29**	0.36**	0.38**	−0.98**	1			
SWC	0.46**	0.49**	0.14	0.14	−0.28**	0.29**	1		
SBD	−0.39**	−0.37**	−0.43**	−0.41**	0.26**	−0.32**	−0.17	1	
pH	−0.49**	−0.49**	−0.02	0.02	0.31**	−0.33**	−0.39**	0.12	1

**Notes:**

** Correlation is significant at the 0.01 level.

SOC, Soil organic carbon (g·kg^−1^); STN, Soil total nitrogen (g·kg^−1^); STP, Soil total phosphorus (g·kg^−1^); STK, Soil total potassium (g·kg^−1^); sand (%); silt (%); SWC, Soil water content (%); SBD, Soil bulk density (g·cm^−3^).

### Relationship between soil nutrients and topographic factors

Topography is one of the main factors influencing soil properties (Moore et al., 1993). Therefore, we performed a correlation analysis between topographic factors (gradient, altitude and slope) and soil nutrients. The slope (°) was transformed into the sine value representing the extent of the eastern slope and the cosine value representing the extent of the northern slope (Lian et al., 2009). Pearson’s correlation coefficient was calculated for each variable to characterize the relationship between soil nutrients and selected topographic factors. The results showed that SOC and STN had a strong positive correlation with Cos a and elevation (*p* < 0.01); STP had a positive correlation with Cos a (*p* < 0.05). Gradient and Sin a were not significantly correlated with other soil nutrients (Table 3).

**Table 3:** Relationship between soil nutrients and topographic factors.

Soil nutrient	Gradient	Cos a	Sin a	Altitude
SOC	0.08	0.35**	0.01	0.28**
STN	0.09	0.34**	0.02	0.27**
STP	−0.08	0.20*	0.02	0.06
STK	0.04	0.03	0.06	0.16

**Notes:**

** Correlation is significant at the 0.01 level.

* Correlation is significant at the 0.05 level (2-tailed).

SOC, Soil organic carbon (g·kg^−1^); STN, Soil total nitrogen (g·kg^−1^); STP, Soil total phosphorus (g·kg^−1^); STK, Soil total potassium (g·kg^−1^); a, slope (°); Cos a, Cosine value of Slope; Sin a, Sine value of Slope; Altitude, m.

### Spatial variability analysis of soil nutrients

We calculated the semi-variogram for all studied variables to characterize the spatial variation of soil nutrients and selected the best models based on the determination coefficient R^2^. The results showed that the reduced sum of squares (RSS) of all nutrients was lower. In addition, the R^2^ of STP was small (0.34), while other R^2^ values ranged from 0.58 to 0.97, which could reflect the spatial heterogeneity of the topsoil nutrients. In most cases, the variograms of the soil nutrients were expressed well by a logarithmic model (STP) or an exponential model (Table 4).

**Table 4:** Parameters of theoretical variogram models for soil nutrients in the Yili Valley.

Soil nutrient	Nuggget	Sill	Range	Proportion	R^2^	RSS	Theoretical
	C_0_	C_0_ + Cs	A_0_ (km)	(Cs/Sill)/%			model
SOC	0.004	0.09	61.0	96.10	0.97	5.48 × 10^−3^	Exp
STN	0.01	0.66	61.0	98.48	0.94	9.88 × 10^−3^	Exp
STP	0.01	0.01	37.6	52.31	0.34	1.38 × 10^−5^	Log
STK	1.08	2.52	61.0	57.22	0.58	0.548	Exp

**Note:**

Cs, Structural variance; R^2^, determination coefficient; RSS, reduced sum of squares; Log, Logarithmic model; Exp, Exponential model.

Nuggets of STK, STP and STN were 1.08, 0.01 and 0.01, respectively. The nugget of STK was 0.004, which indicated a possible error caused by sampling, the experimental process, or the small minimum sample size. The Cs/Sill ratios for the SOC and STN were more than 75%, showing a strong spatial correlation. The Cs/Sill ratios for the STK and STP were between 25% and 75%, which showed that the STK and STP had a medium spatial correlation. The ranges of SOC, STN and STK were all similar, about 61.0 km, while the variation of STP was 37.6 km (Table 4).

### Spatial interpolation of soil nutrients using an Ordinary Kriging interpolation

**Figure 2:**
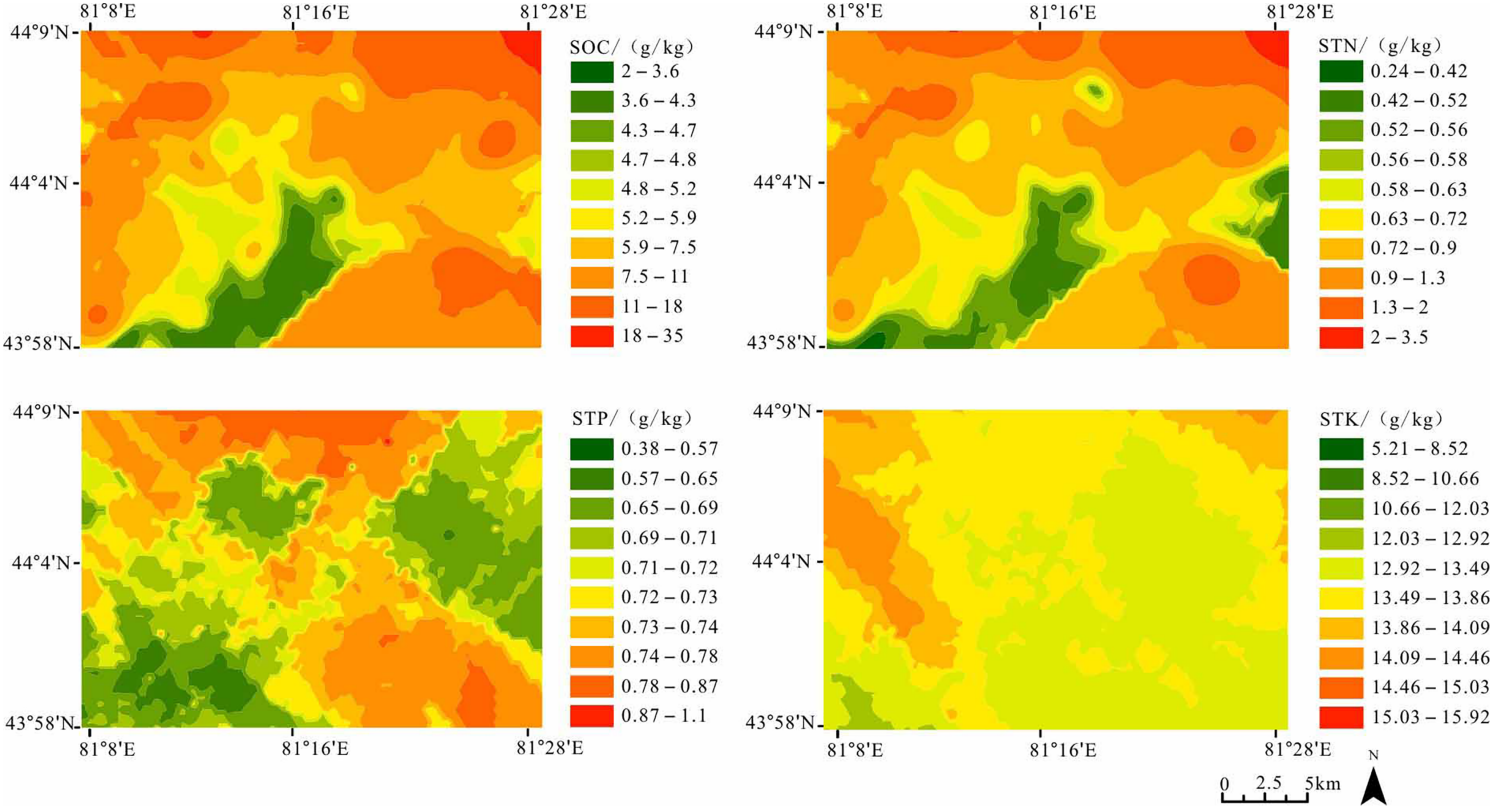
Spatial distribution map of topsoil nutrients in Yili River Valley.

The maps of SOC, STN, STP, and STK using the ordinary kriging interpolation method were drawn. SOC was generally higher in the north than the south with the high-values between 7.5 g·kg^−1^ and 35 g·kg^−1^ distributed in the northwest and northeast, and the low-values between 2 g·kg^−1^ and 4.7 g·kg^−1^ distributed in the middle and southwest. The value of STN was higher in the north between 0.9 g·kg^−1^ and 3.5 g·kg^−1^ than in the central and southwest, where it had a lower value between 0.24 g·kg^−1^ and 0.56 g·kg^−1^. STP was higher in the northwest and southeast (0.74–1.1 g·kg^−1^), and lower in the northeast and southwest (0.38–0.69 g·kg^−1^). The STK was high in the northwest and northeast (14.09–15.92 g·kg^−1^), and low in the central and southeast (5.21–12.03 g·kg^−1^) (Fig. 2).

## Discussion

### Spatial variation characteristics of soil nutrients

Classical statistics showed that in addition to the STK of weak variability, the variation coefficients of SOC, STN and STP were between 12.62–68%, indicating a moderate degree of variation (Table 1). These results were similar to that of other studies (Hu et al., 2007; Wang et al., 2014). Huang et al. (2015) indicated that SOC, TN, TP had moderate variability in a Taklimakan desert highway shelterbelt caused by soil parent materials. A classical statistical analysis can help explain the general status of soil nutrients but cannot effectively reflect their spatial characteristics. Therefore, the semivariance function was used to analyze the spatial variability of soil nutrients. Many studies showed that the spatial variability of soil properties was controlled by intrinsic factors such as soil parent material and soil texture and extrinsic factors such as fertilizer and irrigation application (Yang et al., 2013). The strong correlations of SOC (96.1%) and STN (98.48%) were indicated from the same soil parent material. The moderate spatial correlation of STP (52.31%) and STK (57.22%) indicated that they came from both intrinsic and extrinsic factors (Table 2). In typical soil of karst forest land, Zhang et al. (2013b) found SOC and STN showed moderate spatial correlation. In the oasis of the Manas River Watershed, Maimaiti, Abuduwaili & Ge (2013) found that STN and SOM showed moderate spatial correlation. Our study results differed from previous research (Zhang et al., 2013b; Maimaiti, Abuduwaili & Ge, 2013). These differences may be because our samples are from grasslands, which were often less impacted by human activity and influenced more by natural activities.

The range is an essential parameter for measuring spatial heterogeneity, which is important for sampling space and scope validity. The variable range of SOC, STN and STK were all similar, about 61.0 km, which meant that they had the same ecological process, while the variation of STP was 37.6 km, which showed that the ecological processes of STP were similar. If the sampling grid could map soil nutrient heterogeneity, the sampling grid should be less than the variable range, so the sampling grid of SOC, STN and STK should be less than 60 km and the sampling grid of STP should be less than 30 km.

### Spatial interpolation analysis of soil nutrients

The distributions of SOC and STN were high in the north and low in the south (Fig. 2). Those distributions were mainly influenced by natural factors (topography, elevate and soil texture). Our investigation found that SOC and STN strongly correlate positively with Cos a (*p* < 0.01). We realized that radiation was influenced by the slope—the greater the Cos a, the less the acceptance of solar radiation. In general, the sunny slope was dry, the soil organic matter decomposed rapidly, whereas the shady slope was wet, the accumulation of organic matter was more, and the soil nutrients were relatively high (Lian et al., 2009). Climatic factors also influenced SOC and STN under natural conditions (Sun et al., 2017). SOC and STN have a strong positive correlation with SWC (Table 2). The unique terrain determined less precipitation in plain and more precipitation in the Yili River valley mountainous areas. The increase of precipitation made the vegetation grow vigorously and promoted organic matter accumulation. Sun et al. (2017) argued a significant positive correlation between SOC and precipitation, the same as STN.

We found that SOC and STN have a strong positive correlation with elevation (Table 3). SOC and STN increased with altitude from south to north (Table 3, *p* < 0.01), a result also found by Yang et al. (2010), who hypothesized that SOC and STN increased with elevation. As elevation increases, microbial activity decreases, and the dynamic decomposition rate of animal and plant residues also decreases (Sun, Li & Yang, 2012), causing SOC and STN to increase. STP was high in the northwest and southeast and low in the northeast and southwest. STK was high in the northwest and northeast and low in the central and southeast. STP and STK have no relevance to SWC, pH and elevation, but in a natural environment, STP and STK were influenced by soil parent material more than SOC and STN (Liu, Shao & Wang, 2012).

SOC, STN, STP and STK had a significant correlation with silt content and a negative correlation with sand content and SBD (Table 2). This finding was in agreement with the results of Saggar, who suggested that soil nutrients were often positively associated with fine soil particles (Saggar, Yeates & Shepherd, 2001). SBD is a key physical property of soil that affects the transport of water and solutes, and is essential to assessing nutrient reserves (Yang et al., 2015). SBD generally increased, which means that the process of soil fertility declined (Table 2). Soil texture impacts the input of SOC, STN, STP and STK by influencing vegetation productivity *via* moisture availability and soil fertility (Wang et al., 2010).

## Conclusions

The results showed that the CV values of SOC, STN, STP, sand, silt and SWC displayed a moderate degree of variation. The CV values of STK, SBD and pH indicated a weak degree of variability. The Pearson’s correlation coefficient showed that SOC, STN, STP and STK had a significant correlation with silt content and a negative correlation with sand content and SBD. SWC was significantly positively correlated with SOC and STN, but had no relation to STP and STK. pH was significantly negatively correlated with SOC and STN, but had no relation to STP and STK. SOC and STN have a strong positive correlation with Cos a and elevation. STP has a positive correlation with Cos a. Spatial variability analysis of soil nutrients showed that SOC and STN had a strong spatial correlation, higher values in the north than in the south. STK and STP demonstrated medium spatial correlation. The STK was higher in the northwest and northeast and lower in the central and southeast. STP was higher in the northwest and southeast and lower in the northeast and southwest. The information provided the reference for the rational exploitation of land resources in the Yili River Basin.

## Supplemental Information

10.7717/peerj.13311/supp-1 Supplemental Information 1 Raw data.

## Additional Information and Declarations

### Data Availability

The following information was supplied regarding data availability: The raw measurements are available in the Supplemental File.
